# Management of penetrating cervical injury from needlefish impalement: A case report

**DOI:** 10.1016/j.ijscr.2024.109717

**Published:** 2024-04-27

**Authors:** Putu Anda Tusta Adiputra

**Affiliations:** Department of Surgery, BIMC Siloam Hospital-Kuta, Bali, Indonesia

**Keywords:** Needlefish, Penetrating, Cervical injury

## Abstract

**Introduction:**

Injuries inflicted by needlefish resemble stab wounds, resulting from the rapid and forceful jumping of needlefish jaws from the water's surface. Needlefish impalement on the neck and face are often fatal compared to body areas or extremities. This case report investigates a rare incident where a 50-year-old male tourist sustained a cervical injury from needlefish impalement during an inter-island cruise.

**Case presentation:**

A 50-year-old male experienced right neck pain due to accidental impalement by a needlefish. The initial extraction attempt at a local health center proved unsuccessful, necessitating subsequent surgical intervention. The procedure involved successful removal of the needlefish jaw, wound cleaning, and primary closure with a penrose drain. The patient was discharged on the third postoperative day, showing no neurological deficits or signs of infection during the four-week follow-up.

**Clinical discussion:**

Managing needlefish injuries parallels addressing stab wounds, with the treatment approach guided by the specific location of the injury. Zone III injuries in the neck may pose challenges in bleeding control, especially when involving the internal carotid artery. In this case, a diagnostic approach with computed tomography angiography confirmed vessel integrity, allowing for an exploration approach with an L-shaped skin incision.

**Conclusion:**

Penetrating injuries from needlefish can be life threatening. Treatment strategies must target the injured organ, with consideration of further imaging to assess vascular involvement.

## Highlights

• Penetrating cervical injury from needlefish impalement
• Management of penetrating cervical injury
• Surgical extraction of the needlefish jaw
• Delayed presentation and partial extraction

## 1 Introduction

Bali stands out as the top tourist spot in Indonesia, and the growth of tourism has led to a rise in inter-island human travel. Various water transportation methods are evolving to facilitate movement between islands, heightening the potential for undesired incidents, notably marine animal attacks like those involving needlefish. While such occurrences are exceptionally uncommon, accidents resulting from marine animal encounters, including needlefish incidents, can have serious consequences [1].

Needlefish, a member of the Belonidae family, is identified by its elongated jaw adorned with teeth and a slender body, enabling it to reach speeds of up to 40 miles per hour. These fish typically glide near the water's surface and unexpectedly leap. Injuries inflicted by needlefish resemble stab wounds, resulting from the rapid and forceful jumping of needlefish jaws from the water's surface [2].

There have been limited instances of needlefish impalement reported, with the majority occurring in the Indo-Pacific region. These incidents are predominantly observed among fishermen navigating small boats with lights at night, attracting needlefish. Link et al., documented a case involving a windsurfer. Barss conducted a study reporting 31 cases of needlefish impalement in Papua New Guinea, where attacks predominantly targeted various body areas and extremities [3,4]. A study in Hawaii reported a needlefish impalement on the head and caused brain injury [5]. Clark's study reports needlefish impalement affecting the abdominal area [2]. Another rare case of needlefish impalement of the orbit affect 28 year old patient [6]. Kerkhoffs et al. also reported cases of needlefish impalement in surfers in Portuguese coast [7]. Very rare cases of needlefish impalement are reported in the cervical area. There was one case of a needlefish impalement on the cervical area located in Red Sea region [8].

Needlefish impalement on the neck and face are often fatal compared to body areas or extremities [9]. Due to Bali being a popular tourist destination with extensive maritime activities, the likelihood of needlefish impalement is elevated. However, there have been no documented instances of needlefish impalement affecting the cervical region in Bali to date. This case report will delve into an uncommon occurrence where a tourist sustained a cervical injury from needlefish impalement during an inter-island cruise, successfully resolved through surgical intervention.

## 2 Method

In this case report, we outline the systematic methodology employed in the assessment and management of a 50-year-old male presenting with right neck pain due to accidental impalement by a needlefish. The study adhered to ethical guidelines, and written informed consent was obtained from the patient before commencing the investigation. Comprehensive physical examinations were performed. Diagnostic imaging, including skull x-ray and head CT scan were performed, followed by CT angiography. The patient underwent a surgery to remove the other part of the jaw. The patient's response to treatment was regularly monitored through subjective reports and objective measures, such as neurological deficit or sign of infection. This detailed and systematic approach provides a robust foundation for understanding the management of penetrating cervical injury from needlefish impalement. The work has been reported in line with the SCARE criteria [10].

## 3 Case report

A 50-year-old male tourist, while traveling by boat between Bali and Lombok islands, experienced right neck pain due to accidental impalement by a needlefish. The fish's jaw partially detached during the incident, and the patient attempted extraction at a local health center. Successful removal of one jaw part occurred without significant bleeding. However, the patient was later referred to the hospital after the unsuccessful extraction of the remaining jaw parts.

**Fig. 1:**
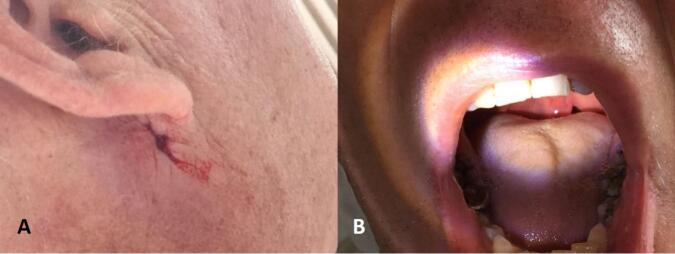
Clinical picture reveals an open wound below the right ear in the right neck.

**Fig. 2:**
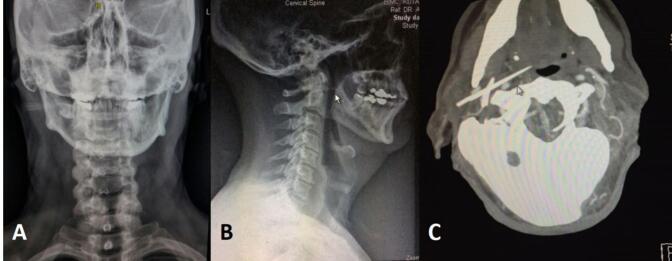
A. Skull AP view; B. Skull lateral view; C. Head CT scan.

**Fig. 3:**
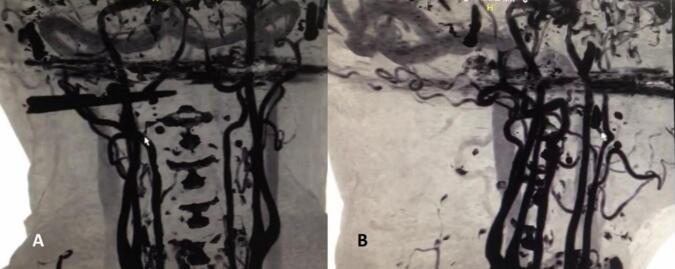
CT Angiography which showed the jaw was struck in the middle of patient's neck vessels between internal and external carotid artery.

In the emergency room, a clinical examination revealed an open wound below the right ear in the right neck (Fig. 1). The primary survey indicated no active bleeding, vocal changes, or dysphagia. Skull x-ray and head CT scan were performed (Fig. 2), followed by CT angiography, which revealed the jaw lodged between the internal and external carotid arteries (Fig. 3).

**Fig. 4:**
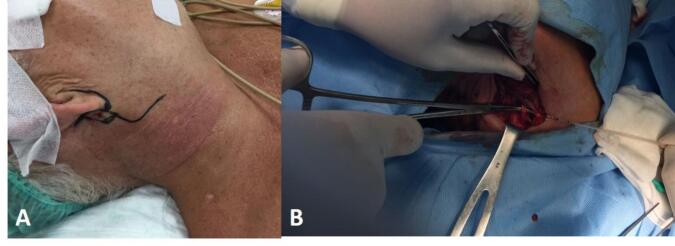
Clinical picture (during the operation). Exploration of the neck revealed the foreign body (jaw) was struck in the middle between external and internal carotid arteries.

**Fig. 5:**
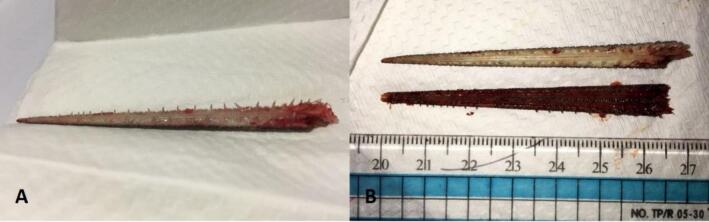
The jaw after extraction.

In the operating room, under general anesthesia, in supine position with head slightly toward to the left side, an L-shaped incision along the angle of the mandible was made. The exploration showed the foreign body (jaw) between the carotid arteries (Figs. 4 and 5). The vessels below were temporarily ligated for controlled bleeding. The jaw was successfully extracted, the wound cleaned, and primary closure with a penrose drain was done. The surgical wound was cleaned with normal saline and primarily closed with penrose drain. Patient was given cefixime antibiotic 200 mg b.i.d for five days post-operative.

Postoperative, patient was stable and fully alert. There's no complain after surgery. The drainage tube was removed on the second day, and the patient was discharged on the third day after the surgery. After four weeks of follow up, there was no neurological deficit or sign of infection.

## 4 Discussion

The needlefish belongs to the Belonidae family, with “belone” deriving from the Greek word for needle [9]. Their method of hunting and evading larger predators involves leaping and skimming across the water's surface. The majority of injuries are believed to be unintentional, resulting from startling the needlefish, rather than a deliberate attack [11]. Needlefish induce penetrating injuries without injecting venom. The combination of the needlefish's speed and distinctive shape is ideal for causing severe penetrating wounds. The elongated snout can reach internal organs, resulting in significant damage. Following skin penetration, the jaw may fracture into multiple fragments, causing serious harm at a distance from the initial entry point [12].

Managing this injury is akin to addressing a stab wound, and the approach is primarily guided by the injury's specific location. Penetrating neck wounds, especially those potentially affecting cervical vascular structures, have been categorized into three distinct zones since their initial description by Monson et al. in 1969 [13].

Zone I extends below the clavicles and the manubrium sterni, encompassing all structures within the thoracic outlet. Zone II lies between the thoracic outlet and the angle of the mandible, while Zone III spans between the angle of the mandible and the base of the skull. Given that the wound's tip is just beneath the patient's right ear, our patient falls into Zone III of neck injury [14].

Patients with penetrating or blunt injuries to the three zones of the neck present with overt symptoms or signs, moderate or modest symptoms or signs, or they are asymptomatic without signs of aerodigestive or vascular injury. When overt symptoms and/or signs are present, standard “ABC” resuscitation as described in the ATLS manual is performed. With moderate or modest symptoms or signs, a variety of diagnostic tests including cervical CT, conventional arteriography, duplex ultrasonography, color flow Doppler, CTA, esophagography, and fiber-optic esophagoscopy, tracheoscopy, and bronchoscopy are used to determine whether an injury to the carotid artery system, vertebral arteries, esophagus, or trachea is present [14].

There are major blood vessels and cranial nerves close to the base of the skull in the Zone III. Patients with arterial injuries in this zone may be asymptomatic at the beginning, and control of bleeding in this zone may be quite difficult [13]. The patient had one needlefish jaw extracted at the local health center, but the procedure lacked diagnostic support, potentially leading to fatal consequences.

Imaging studies also recommended determining the presence of a foreign body (i.e., retained jaw) and the degree of internal injury. The jaw is usually radio-opaque (as are the spines of stingrays and catfish) [15], which had showed in our cervical CT scan. The jaw was radioopaque and struck in the middle of external and internal carotid arteries. Arteriography can be helpful if the patient is stable. Immediate surgery to explore wounds and repair injuries is recommended if arteriography is not feasible and vascular injury is suspected [16]. We did the CT angiography and showed that vessels were intact.

In this patient, CT scan was initially performed to evaluate the involvement of soft tissues that might be affected, such as the trachea, and so on. However, due to its location in the neck and suspicion of involvement with the jugular vein, a CT angiography was conducted. The CT scan was performed at two different times and locations because at the first hospital visited by the patient, only a CT scan without CT angiography could be performed. Therefore, it is necessary to repeat both the CT scan and CT angiography at a different hospital.

For Zone III injuries, surgical access typically involves temporomandibular joint subluxation or vertical ramus mandibulotomy to expose the distal internal carotid artery. However, since CTA confirmed vessel integrity, we chose an exploration approach with an L-shaped skin incision from beneath the right ear to the angle of the mandible, revealing the proximal end of the vessels.

There is another similar case reported by Ali et al. [17], involving a 20-year-old Maldivian male who was impaled by a needlefish. The patient sustained a penetrating injury to the right side of the neck. Initial assessment at the local clinic revealed no evidence of vascular injury. The patient was empirically treated with ceftriaxone and metronidazole, along with analgesia and antitetanus toxoid (ATT) injection. CT imaging showed a 3-cm foreign body that avoided vital neck structures. After confirmation that no major structures were involved, the patient underwent wound exploration under general anesthesia. The wound, located in the posterior triangle of the neck, was left open to heal by secondary intention. The patient was discharged with oral antibiotics and made a complete recovery.

Another case involved a 53-year-old female who was penetrated in zone II of the neck while swimming. Cervical radiograph revealed a radioopaque foreign body. CT scan showed a 3.7 cm calcified foreign body. The patient underwent neck exploration and primary wound closure with a drain under general anesthesia. Intravenous cefazolin was administered to the patient [8]. In another case, a 20-year-old male was impaled in zone III of the neck while surfing. Radiologic examination did not show any perforation or bone lesion. Manual removal using straight Kelly clamps was performed under local anesthesia. The patient was prescribed oral cephalexin for antibiotic treatment [19].

In the three aforementioned cases, the management provided to this patient has been deemed appropriate, showcasing a comprehensive approach to addressing needlefish impalement injuries. These cases underscore the significance of conducting thorough examinations and employing various diagnostic modalities, such as CT scans and angiography, to assess the extent of injury accurately. The utilization of CT imaging not only aids in identifying the presence of foreign bodies but also assists in evaluating potential damage to surrounding structures, including major blood vessels and nerves.

Furthermore, the described management protocol involves meticulous surgical intervention, wherein the removal of the foreign body and repair of any associated injuries are performed under general anesthesia. This approach ensures optimal patient care and minimizes the risk of complications associated with penetrating cervical injuries. Moreover, the administration of antibiotics postoperatively serves to mitigate the risk of infection and promotes the patient's recovery process. The choice of cefixime antibiotic highlights the importance of selecting appropriate antimicrobial agents based on individual patient characteristics and the anticipated microbial profile.

Overall, the successful outcomes observed in these cases underscore the effectiveness of the multidisciplinary approach adopted in managing needlefish impalement injuries. The meticulous attention to detail, coupled with prompt and appropriate interventions, has contributed to the favorable prognosis and eventual discharge of the patients in good condition. The limitation of the study is the article focuses on a single case study and may not be applicable to all cases of needlefish impalement. The findings and management approach may not be generalizable to cases with different circumstances or clinical presentations. Which limits the ability to draw broad conclusions or establish statistical significance.

## 5 Conclusion

Penetrating injuries from needlefish can be life threatening. Treatment should be specified at the organ injured, especially in the cervical area. Further imaging should be considered for possibility vascular involvement as extent of the injury

## Informed consent

Written informed consent for the case report publication was obtained from the patient.

## Ethical approval

The article has been reviewed by Hospital Legal and Ethics Committee.

## Funding

None to declare. This research did not receive any funding from any institution, or funding agencies.

## Author contribution

The conception, design, execution, and interpretation of the entire study.

## Guarantor

Putu Anda Tusta Adiputra

## Conflict of interest statement

None to declare.
